# Does Lymphovenous Anastomosis Effect Mammalian Target of Rapamycin Inhibitor-associated Lymphedema Patients?

**DOI:** 10.1055/a-2201-5881

**Published:** 2024-03-04

**Authors:** Inah Yoon, Hyung Bae Kim, Jeongmok Cho, Changsik John Pak, Hyunsuk Peter Suh, Jae Yong Jeon, Joon Pio Hong

**Affiliations:** 1 Department of Plastic and Reconstructive Surgery, Asan Medical Center, University of Ulsan College of Medicine, Seoul, Korea; 2 Department of Rehabilitation Medicine, Asan Medical Center, University of Ulsan College of Medicine, Seoul, Korea

**Keywords:** mTOR inhibitors, sirolimus, lymphedema, immunosuppressive agents, lipectomy

## Abstract

The mammalian target of rapamycin (mTOR) inhibitors are used to prevent organ transplant rejection and are preferred over other immunosuppressants due to its low nephrotoxicity. However, mTOR inhibitors have been associated with various adverse effects including lymphedema. Although rare in incidence, previously known treatments for mTOR inhibitor-induced lymphedema were limited to discontinuation of related drugs and complex disruptive therapy with variable results.

In this article, three patients who developed lymphedema in their lower limbs after using mTOR inhibitors, including two bilateral and one unilateral case, were treated with physiologic surgery methods such as lymphovenous anastomosis (LVA) and lymph node transfer. The efficacy of the treatment was evaluated.

In the three cases described, cessation of the drug did not lead to any reduction in edema. The use of LVA and lymph node transfer resulted in early reductions in volume but failed to sustain over time. All patients underwent secondary nonphysiologic surgery such as liposuction resulting in sustained improvement.

This series presents the first physiologic approach to mTOR inhibitor-induced lymphedema. Although further study is warranted, the physiologic surgical options may have limited success and nonphysiologic options may offer better sustainable results.

## Introduction

The mammalian target of rapamycin (mTOR) inhibitors, such as sirolimus and everolimus, are used to prevent organ transplant rejection and minimize side effects such as infection. 1 2 The mTOR inhibitors have advantages of low nephrotoxicity compared with other immunosuppressants, but various adverse effects have been reported including thrombocytopenia, dyslipidemia, delayed wound healing, and in rare cases, lymphedema. In patients treated with mTOR inhibitors, lymphedema has been reported to have prevalence of 6 to 12%. 3 4

The mTOR inhibitor inhibits serine/threonine kinase involved in cell growth, including synthesis and metabolism of fat and protein, and proline-rich protein kinase B signaling involved in cell survival and angiogenesis. In particular, it downregulates vascular endothelial growth factor (VEGF) involved in angiogenesis, which also inhibits VEGF C and VEGF D, which are required for lymphatic formation, and consequently suppresses lymphangiogenesis. 3 5 6 Further research showed that lymphatic vessel endothelial receptor was less expressed, lymphatic drainage was slower, and collateral vessel formation was less in mTOR inhibitor-treated mice than in the control group. 7

Despite the concern of mTOR inhibitor-induced lymphedema, there has only been a handful reports on extremity lymphedema related to sirolimus. 4 5 8 9 10 Furthermore, the treatment for mTOR inhibitor-induced lymphedema has been focused on cessation of drugs and provide complex decongestive therapy (CDT). 1 11 To the best of authors' knowledge, there has been no report and is the first study using the physiologic surgical approach for these patients.

This paper describes three patients with five lower limbs lymphedema (two bilateral and one unilateral) induced after mTOR inhibitor use. These patients are approached by physiologic surgery such as lymphovenous anastomosis (LVA) and lymph node transfer as first-line and secondary liposuction when physiologic surgery was not beneficial. 12

## Cases

Between 2014 and 2022, three patients with mTOR inhibitor-associated lymphedema after transplantation had undergone lymphedema surgery such as LVA, liposuction, or lymph node transfer at single center ( Table 1 ).

**Table 1:** Patient characteristics

Patient	Age/Sex	Transplant/Site/Indication	Immunosuppressant	Duration of SRL/EVL up to onset	Additional SRL/EVL period after onset	Dosage	Site of lymphedema	Lymphoscintigraphy	Other factors
1	45/M	Renal/Right iliac fossa/ESRD due to unknown cause	Sirolimus, tacrolimus, myocophenoic acid	102 months	21 months	1 to 5 mg	Scrotum, Both LL	Both LL: failure of superficial collectors	Secondary HTN, both femoral head AVN
2	47/M	Renal/Right iliac fossa/ESRD due to glomerulonephritis	Sirolimus, tacrolimus, methylprednisolone	12 months	60 months	1 to 3 mg	Right LL	Right LL: failure of superficial collectors	Taking synthyroxine due to history of thyroidectomy
3	75/M	Heart/Chest/DCMP	Everolimus, cyclosporin, myocophenoic acid	42 months	0 month	0.5 to 0.75 mg	Both LL	Both LL: reduced superficial collectors and delayed lymphatic drainage	None

Abbreviations: AVN, avascular necrosis; CDT, complex decongestive therapy; DCMP, dilated cardiomyopathy; ESRD, end-stage renal disease; HTN, hypertension; LL, lower limb; SRL/EVL, sirolimus/everolimus.

### Patient 1

A 45-year-old man, who underwent renal transplantation due to unknown cause of end-stage renal disease (ESRD), developed progressive (both legs and scrotal) lymphedema 10 years after transplantation surgery. He had no family history of lymphedema, spared lymph nodes, no radiation, no infection, no cancer lesion, and the only relevant factor for lymphedema formation was sirolimus taken for 102 months (1–5 mg daily). After the aggravation of lymphedema for 21 months, he stopped taking sirolimus which had minimal effect to reduce lymphedema.

**Fig. 1:**
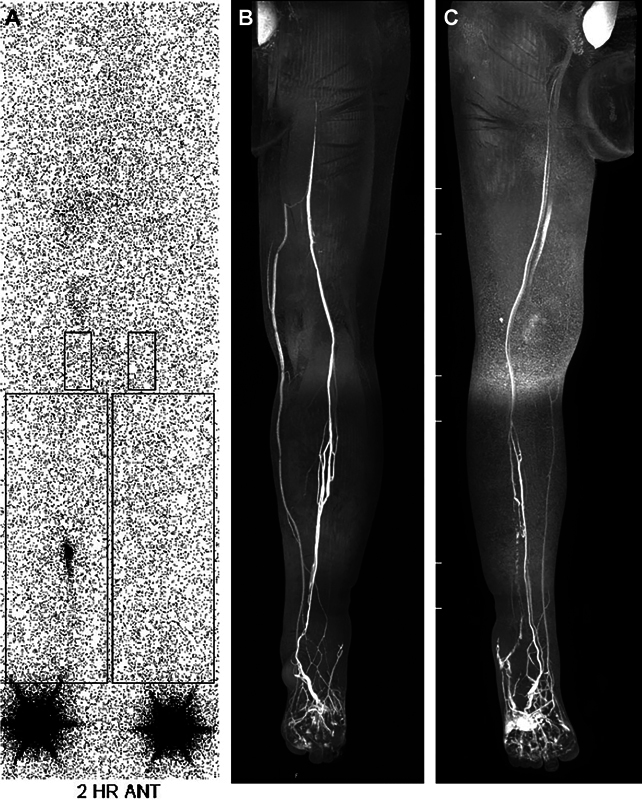
( **A** ) Preoperative lymphoscintigraphy, 2-hour delayed image. ( **B** ) Preoperative MR, lower extremity, left leg. ( **C** ) Preoperative MR, lower extremity, right leg.

**Fig. 2:**
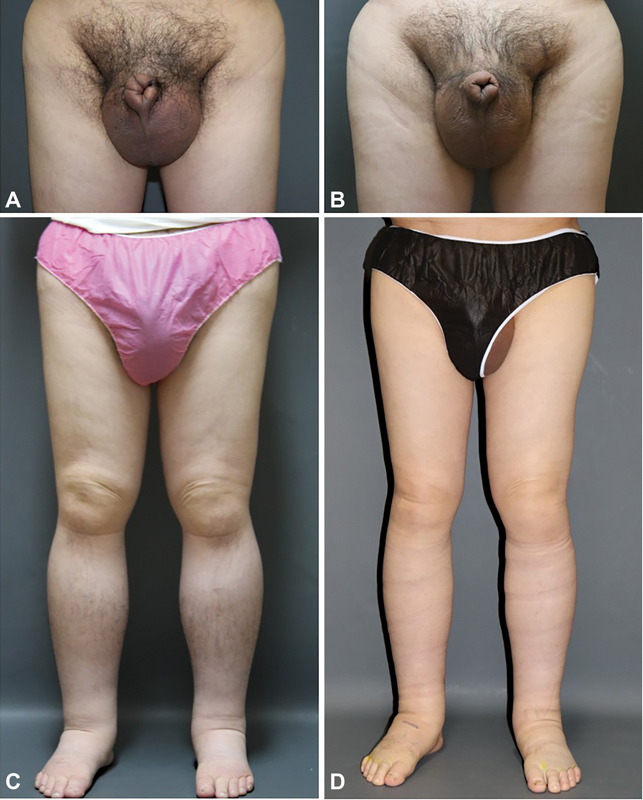
( **A** ) Preoperative clinical photography of the patient 1, scrotum. ( **B** ) Postoperative clinical photography of the patient 1, scrotum. ( **C** ) Preoperative clinical photography of the patient 1, both legs. ( **D** ) Postoperative clinical photography of the patient 1, both legs.

Lymphoscintigraphy demonstrated lymphedema in both lower extremity with nonvisualization of both inguinal lymph node on 2-hour delayed image. There was no definite visible lymphatics in the left leg on magnetic resonance lymphangiogram ( Fig. 1 ). Although 8 months after discontinuation of sirolimus, his lymphedema persisted and aggravated ( Fig. 2A, C ).

The patient underwent LVA surgery on four sites in both extremities. Three of the lymphatic vessels in both legs were too sclerotic, and only one LVA could be performed. One site in the right upper scrotal area was suitable for LVA and was successfully performed. The diameter of the lymphatic vessel was 0.6 mm, and the vein size was 0.6 mm. Side-to-end anastomosis was performed between the lymphatic vessel and vein. The shunting flow after the anastomosis was fast and there was no backflow. The spermatic cord was preserved. Immediately after LVA, the patient's scrotum volume decreased rapidly. The patient reported that his genital area was very light and the redness of scrotum was reduced. Immediately after the surgery, both legs showed a decrease in the circumference for 6 months but started to recur after 1 year ( Fig. 2B , D, Table 2 ).

**Table 2:** Outcomes after surgery

Patient	Side of limb	Measurement timing (m, month; y, year)	Circumference						Volume (cm ^3^ ) a	Body mass index
			Above knee 15 cm	Above knee 10 cm	Above knee 5 cm	Below knee 5 cm	Below knee 10 cm	Below knee 15 cm		
1	Rt.	Pre	46.0	42.5	38.8	36.5	39.5	40.5	4,473.93	25.90
		POD 1 m	46.2	41.8	38.5	35.2	37.4	38.0	4,246.85	25.35
		POD 6 m	46.0	43.0	39.0	34.7	37.3	37.0	4,129.78	25.00
		POD year	48.0	45.5	41.5	35.5	40.0	40.0	4,636.94	25.30
	Lt.	Pre	49.0	44.5	40.0	37.5	41.0	41.0	4849.52	–
		POD 1 m	47.8	43.5	39.5	36.0	39.0	39.0	4,514.36	–
		POD 6 m	48.5	43.0	41.0	37.5	39.0	38.0	4,489.85	–
		POD year	47.0	43.5	41.0	35.0	39.0	39.5	4,479.10	–
2	Rt.	Pre	55.0	51.9	47.2	44.5	46.2	45.8	6,084.11	23.65
		POD 1 m	55.0	51.0	47.0	40.0	42.0	42.0	5,652.07	23.32
		POD 6 m	53.0	49.0	44.5	42.5	43.5	44.0	5,634.55	23.45
		POD year	56.6	53.1	49.4	42.3	45.1	43.5	6,017.44	24.44
		POD 2 y	57.2	54.5	48.5	47.5	48.5	49.0	6,748.12	24.77
		POD 3 y (preliposuction)	52.7	50.0	45.0	43.2	46.6	47.0	5,942.03	24.44
		Postliposuction 1 m	48.1	44.3	42.1	38.2	40.8	40.1	4,657.99	23.24
		Postliposuction year	52.0	46.0	44.5	39.0	39.0	38.5	4,926.95	24.42
3	Rt.	Pre	58.0	57.0	–	48.0	46.5	45.0	6,729.30	30.33
		POD 6 m	51.5	49.0	–	47.0	45.5	45.0	5,797.57	30.56
		POD year	59.5	55.0	–	46.0	45.0	43.2	6,682.52	31.24
		POD 2 y	64.0	58.0	–	55.0	54.5	58.5	8,472.13	31.77
	Lt.	Pre	59.0	57.0	–	49.0	48.5	45.0	6,984.87	–
		POD 6 m	51.0	50.0	–	47.0	45.5	45.0	5,738.06	–
		POD year	56.0	56.0	–	46.0	44.6	45.0	6,232.48	–
		POD 2 y	62.0	58.0	–	57.0	54.0	53.0	8,460.99	–

Abbreviations: π, constant; h, height; Lt., left; R, radius on base; r, radius on top; Rt., right; POD, post operative day.

V = π × h × ( *R* ^2^ + *r* ^2^ + *Rr* )/3

a The volume segment was measured according to the formula of a truncated cone.

### Patient 2

A 47-year-old man underwent kidney transplantation due to ESRD, and lymphedema in the right leg began 2 years after surgery especially taking sirolimus for the last 12 months. No other cause was suspected other than the drug taken. Lymphoscintigraphy confirmed lymphedema of his right leg. The patient underwent CDT without improvement. The patient underwent LVA surgery on his right lower leg. Four sites of LVAs were performed on the patient's right foot dorsum. Sirolimus was stopped following the LVA surgery. Initial progress showed slight improvement but recurred after 1 year despite CDT. Secondary nonphysiologic approach using liposuction was performed 3 years after LVA surgery. There was initially 22% reduction of volume at 1 month and maintained approximately 17% reduction of volume with no recurrence of cellulitis at 1 year after nonphysiologic surgery and CDT ( Table 2 ).

### Patient 3

A 75-year-old man who had undergone heart transplantation due to dilated cardiomyopathy with heart failure started taking everolimus 0.25 mg/day. After 42 months of taking everolimus, discomfort and swelling on both lower legs started. The patient had no other factors for lymphedema and was suspected with drug-induced lymphedema. Despite cessation, lymphedema worsened, and diagnosis was confirmed by lymphoscintigraphy. The CDT was not helpful in reducing the swelling. Initially, LVA was planned but exploration showed complete sclerotic lymphatic vessels and LVA was not able to be performed. As per surgical algorithm, a physiologic approach using two 6 × 2.5 cm-sized supraclavicular vascularized flap with lymph node were harvested on both supraclavicular posterior triangular area and transplanted on both ankles. 13 14 At 6 months, the patient showed reduction in volume for both legs. Unfortunately, the lymphedema recurred at 1 year and further aggravated despite CDT. The patient's lymphedema was aggravated and suffered refractory cellulitis during the follow-up of 2 years. The patient was offered nonphysiologic approach but refused further surgery.

## Discussion

Despite the reports on mTOR inhibitor-induced lymphedema, a clear understanding of mechanism remains to be obscure and treatment limited to cessation of the drug and to provide CDT. 3 7 12 15 According to some reports, the benefit of stopping the mTOR inhibitor remains controversial. 5 9 One of them reports reflecting the improvement based on duration used (usually within 7–30 months are mainly reversible). 3 In this study, until the onset of symptoms, each patient took 102, 12, and 42 months of mTOR inhibitor, respectively. In addition, all of them stopped taking drugs, but there was no effect in edema reduction.

Since transplant patients continue to take immunosuppressive drugs for life, a need for a therapeutic approach to treat lymphedema is required rather than simply stopping the essential drug. Thus, three lymphedema patients (five lower limbs) induced secondarily by mTOR inhibitor underwent physiologic surgical approach to improve the symptoms. In the three limbs that were first approached with LVA on the limb, only one limb (patient 2) had initially a functional lymphatic vessel leading to successful lower limb LVA. The other patient (patient 1 with bilateral lower limb and genital lymphedema) did not have good functioning lymphatic vessels on the limb and functional lymphatic vessels were only found near the genitalia leading to successful LVA. The patient 3 with bilateral lower limb lymphedema patient undertook lymph node transfer due to sclerotic lymphatic vessels. These two patients have prolonged use of mTOR inhibitor in common with at least 42 months. Further investigation is warranted to evaluate the relationship of lymphedema formation against the duration of usage. Another factor that needs further investigation is why some patients develop lymphedema while the majority of the patients with prolonged use of mTOR inhibitor do not. The rarity of these cases makes it difficult to evaluate such factors.

**Fig. 3:**
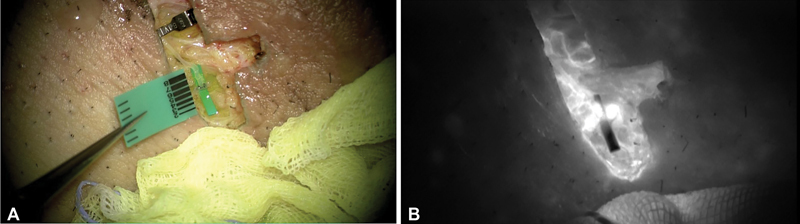
( **A** ) Lymphovenous anastomosis, intraoperative microscopic image. ( **B** ) Lymphovenous anastomosis, post-indocyanine green image.

When LVA was performed for patients 1 and 2, the patency of the anastomosis was confirmed under intraoperative, indocyanine green (ICG—dye; 16 17 Fig. 3 ). The initial effect after LVA was further supported by reduction of genital size and limb volume, which had not responded to CDT for 2 years prior to surgery. In the case with lymph node flap, the follow-up at 6 months also showed reduction of volume supporting initial benefits from this approach as well. In all three cases, the volume of the affected five limbs and the genitalia decreased by approximately 9 to 16% during the first 6 months. However, despite the continuous use of the same CDT protocol and discontinuation of the mTOR inhibitor, it gradually increased back to the presurgical volume or even worsened in four limbs ( Table 2 ). It can be speculated that initial effect from physiologic surgery may work toward reducing the volume but only temporarily. Although histologically, confirmation is needed, the authors suspect that the sclerosis from mTOR inhibitors may exert a different effect on lymphatics compared with simple obstruction-induced sclerosis, thus limiting the effect only temporarily.

In one patient where secondary liposuction was performed, the limb volume was reduced and maintained well for the duration of follow-up with CDT. The reason for this favorable response can be speculated based on mTOR inhibitor effect involved in fat synthesis and metabolism that causes dyslipidemia leading to lipedema. 3 Kim et al also said that mTOR inhibitor-induced lymphedema showed a higher subcutaneous fat ratio in lower extreme CT venography. 2 Furthermore, the aggravation after temporary improvement from the physiologic surgery may be partially explained by this mechanism as well. Currently, we can use bioimpedance analysis to accurately measure the fluid contents on the affected limb. 18 However, during the time of this case, it was not available. Thus, it was difficult to evaluate the efficacy of physiologic surgery and the effect it has on further aggravation after surgery. Further evaluation and histologic examination may be needed in the future to confirm the effect of physiologic surgery.

Also, the use of novel imaging modalities such as near-infrared fluorescence lymphography, ultrasound imaging, and photoacoustic imaging can potentially improve the effectiveness of physiologic surgery. 19 20 Instead, in this study, immediate volume reduction and successful LVA were confirmed through intraoperative ICG and microscopic imaging assessments of lymphatic patency and shunting flow. However, despite initial positive outcomes, long-term follow-up revealed a recurrence of lymphedema. Therefore, although effective imaging modalities may enhance the success rate of physiologic surgery, their impact on surgical outcomes in mTOR inhibitor-induced lymphedema remains uncertain.

This is the first report using physiologic surgery against mTOR inhibitor-induced lymphedema. There are three major findings based on this case report. First, the lymphatic vessels of mTOR inhibitor-induced lymphedema patients may have degenerated and nonfunctioning lymphatic vessels based on the duration of drug use. Second, when LVA is successful, early reduction of volume was only temporary. Third, liposuction after successful LVA may have implications for a more lasting improvement. Although further studies are warranted to see the effects of physiologic surgery against mTOR-induced lymphedema, due to the scarcity of mTOR inhibitor-induced lymphedema, this report of three patients (five limbs and one genital lymphedema) may shed early light into a difficult problem.

In conclusion, this series presents the first physiologic approach to mTOR inhibitor-induced lymphedema. Although further study is warranted, the physiologic surgical options may have limited success and nonphysiologic options may offer better sustainable results.
